# Cost savings associated with an MRI based strategy in suspected severe mitral regurgitation

**DOI:** 10.1186/1532-429X-18-S1-O134

**Published:** 2016-01-27

**Authors:** Seth Uretsky, Edgar Argulian, Hira Awan, Ashadevi Jagarlamudi, Steven Wolff

**Affiliations:** 1 grid.416113.00000000097594781Cardiovascular Medicine, Morristown Medical Center, Morristown, NJ USA; 2 Medicine, Mount Sinai St. Luke's Hospital, New York, NY USA; 3 Radiology, Carnegie Hill Radiology, New York, NY USA

## Background

Recent studies have shown that echocardiography and MRI are often discordant when assessing the severity of mitral regurgitation (MR), with echocardiography often overestimating severity of mitral regurgitation compared to MRI (1). This is particularly true among patients who underwent mitral valve surgery based on echocardiographic assessment and the current ACC/AHA recommendations for valvular disease. These findings not only have potential impact on patient management it also has an impact on the healthcare costs associated with mitral valve surgery.

## Methods

We prospectively enrolled 115 (61 ± 14 yrs, male 67%) patients with MR in a multicenter trial that underwent evaluation with both echocardiography and MRI. Each echocardiogram and MRI study was assessed for the severity of MR. Appropriateness of surgery was determined based on the ACC/AHA recommendations. The cost of mitral valve surgery was estimated conservatively as $30,000 based on prior publications (2). The cost of echocardiography ($264.48) and MRI ($431.80) was based on Medicare part B reimbursement.

## Results

Among the 115 patients, 40 (35%) patients underwent indicated mitral valve surgery based on echocardiographic evaluation of MR severity and the ACC/AHA guidelines, with 93% having a class I indication and 7% having a class IIa indication. Of the 40 patients who underwent mitral valve surgery, 14 (35%) had a class I indication by MRI and 26 (65%) did not have an indication for mitral valve surgery. Based on cost estimates, an MRI based strategy would of resulted in a savings of $762,728 in our study cohort (Table 1).

## Conclusions

In patients referred for mitral valve surgery based on echocardiographic assessment and ACC/AHA guidelines, an MRI based strategy would have resulted in a cost savings of more than $19,068 per patient. This estimate is conservative because it does not include costs related to surgery, such as patient morbidity or the need for long term anticoagulation and monitoring. According to a recent study (3), there are approximately 5,890 isolated mitral valve surgeries per year for patients with mitral regurgitation. Based on these findings, an MRI based strategy applied to all of the US could potentially result in a cost savings to the healthcare system of more than $100 million annually.

**Table 1:** Cost differences between an echocardiography based strategy vs. an MRI based strategy

	Echocardiography based strategy	MRI based strategy
Echocardiography	$10,739.20	$10,739.20
MRI		$17,272.00
Surgery	$1,200,000	$420,000
Total Costs	$1,210,739.20	$448,011.20
